# Graphene Oxide-Functionalized Optical Sensor for Label-Free Detection of Breast Cancer Cells

**DOI:** 10.1021/acsanm.5c02864

**Published:** 2025-08-18

**Authors:** Jiaxing Sun, Hanlin Jiang, Kartikey J. Chavan, Amanda S. Coutts, Xianfeng Chen

**Affiliations:** † Department of Physics, School of Science and Technology, 6122 Nottingham Trent University, Nottingham NG11 8NS, United Kingdom; ‡ John van Geest Cancer Research Centre, Department of Biosciences, Nottingham Trent University, Nottingham NG11 8NS, United Kingdom

**Keywords:** Graphene oxide, Long-period fiber grating, Label-free sensor, Cancer cell detection, Breast cancer

## Abstract

Accurate and noninvasive detection of cancer cells is critical for advancing early stage cancer diagnostics and monitoring tumor progression. While manual enumeration methods, such as hemocytometry, remain in use, they suffer from limited sensitivity and scalability. In this article, we report the first feasibility study demonstrating a graphene oxide (GO)-functionalized long-period fiber grating (LPG) sensor for the label-free detection of MCF-7 human breast cancer cell density via secreted cellular byproducts. The sensing mechanism is based on refractive index (RI) modulation in the culture medium, where the GO overlay serves as a functional interface to enhance light–matter interaction and mode coupling between the LPG device and the external medium. GO nanocoatings were deposited on the device surface via an in situ layer-by-layer (i-LbL) assembly method and characterized using scanning electron microscopy (SEM), atomic force microscopy (AFM), and Raman spectroscopy. Furthermore, by precisely controlling the thickness of the GO nanocoating, we experimentally investigated the impact of the GO thickness on the optical properties, revealing distinct thickness-dependent behavior. Resonance changes correlated clearly with metabolite accumulation, thus enabling indirect detection of cancer cell density. The GO-LPG sensor demonstrated detection of MCF-7 cell densities ranging from 0 to 1 × 10^5^ cells/mL, achieving ultrahigh sensitivity with a limit of detection (LOD) as low as 270 cells/mL. This GO-functionalized fiber optic configuration offers significant potential as a real-time, label-free, and noninvasive bionanophotonic platform for cancer diagnostics and metabolic sensing in complex biological environments.

## Graphical abstract

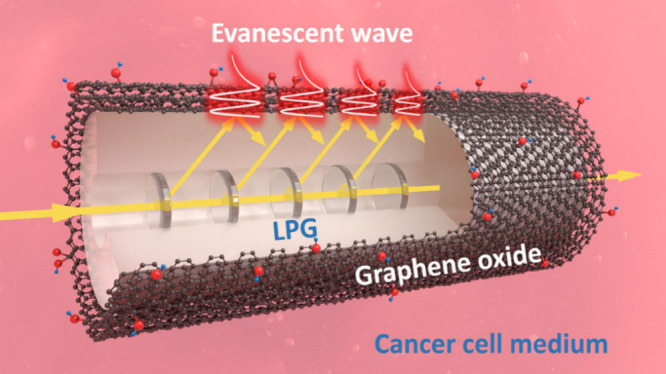

## 1 Introduction

Cancer cells exhibit distinct metabolic characteristics compared to normal cells, notably the Warburg effect, where glycolysis remains the predominant energy production pathway even in the presence of oxygen.^1^ These metabolic profiles not only offer early indicators of malignancy but also provide insight into tumor biology, enabling the development of personalized therapeutic strategies and facilitating treatment monitoring.^2,3^ It is well-known that cells consume nutrients and release metabolites into their culture medium during growth incubation. Cell metabolites are small molecules involved in or produced by metabolic processes, such as amino acids, sugars, lipids, and energy-related compounds. Changes in these metabolites can reflect the physiological state of the cancer cells. Their concentrations in culture media change with cellular activity and quantity, making them attractive targets for noninvasive cancer diagnostics.^4,5^ Dulbecco’s Modified Eagle’s Medium (DMEM), widely used in cell culture, mimics the in vivo environment by supplying essential nutrients for cellular proliferation. As the cell number increases, metabolic activity alters the chemical composition of the medium. Conventionally, cell counting is performed manually by using a hemocytometer prior to incubation. While alternative techniques such as mass spectrometry and nuclear magnetic resonance spectroscopy have been employed to profile these metabolites, they often involve complex sample preparations, lengthy analysis times, and high operational costs.^6^ To address these limitations, optical biosensors have emerged as promising platforms for rapid, cost-effective, and label-free detection of metabolic variations in live cancer cell cultures.^7–11^

Graphene oxide (GO), a derivative of graphene, has drawn great attention due to its large π-conjugated planar structure and abundance of oxygen, epoxy, and hydroxyl groups on the basal plane and carboxyl groups at the edges.^12–16^ These features endow GO with excellent liquid dispersibility, biocompatibility, and surface modifiability, making it suitable for biomedical applications. So far, GO has been explored in various applications, including drug delivery,^17,18^ photothermal therapy,^19,20^ and the detection of glucose,^21^ hemoglobin,^22^ cortisol,^23^ protein,^24^ cancer cells,^25^ microRNA,^26^ antibodies,^27^ and DNA.^28^ Over the past few decades, fiber optic technologies have advanced through the development of fiber gratings, fiber optic Fabry–Perot interferometers, surface plasmon resonance fiber sensors, and tapered fibers.^29–32^ These technologies offer advantages such as label-free operation, multiplexing capability, and rapid detection, making them promising tools for early cancer diagnostics^29–32^ and disease detections.^33–36^ Functionalization of long-period fiber gratings (LPGs) with GO coatings has further enhanced the sensing performance.^24,27,37^ Depending on the applications, GO coatings have varied from a thicker overlay (hundreds of nanometers to several micrometers) for humidity or hemoglobin sensing^22,38,39^ to thinner coatings (∼50 nm) for gas or immunosensing.^27,40^ It has been reported that the thickness of the coating material plays a critical role in optical sensors, as an increased layer can hinder the evanescent field from effectively penetrating the coating, thereby affecting sensor performance.^41,42^ The optical properties of GO are critical to next-generation nanophotonic devices.^43^ However, research investigating the effect of GO thickness on optical performance has been limited mainly due to the lack of efficient methods for precisely controlling and determining nanocoating thickness.^14^

Accurate and label-free detection of cancer cells remains a key challenge for early stage diagnostics and effective tumor monitoring. In this article, we report a first-of-its-kind feasibility study demonstrating a GO-functionalized LPG sensor for label-free detection of MCF-7 human breast cancer cell density by monitoring refractive index (RI) changes of the culture medium induced by cellular byproducts. As the schematic illustrated in Figure 1 shows, the LPG couples the light from the fiber core to the cladding to act as an optical transducer, while the GO overlay serves as the light–matter interface between the optical device and the surrounding medium. GO-LPG detects RI changes in the culture medium, which correlate with the number of cancer cells via their secreted metabolites. GO overlays were deposited on the LPG using an in situ layer-by-layer (i-LbL) assembly technique to achieve precisely controlled thicknesses (55.1 nm, 125.2 nm) via the tunable deposition parameters including the number of coating cycles, GO nanosheet concentration, and solvent evaporation time. Furthermore, taking advantage of the precisely controlled thickness, we experimentally investigated the influence of GO thickness on optical properties. The GO-LPG sensor was used for label-free detection of MCF-7 cancer cell density via culture medium, offering a promising bionanophotonic interface for applications in biomedical diagnostics and early cancer detection.

**Figure 1:**
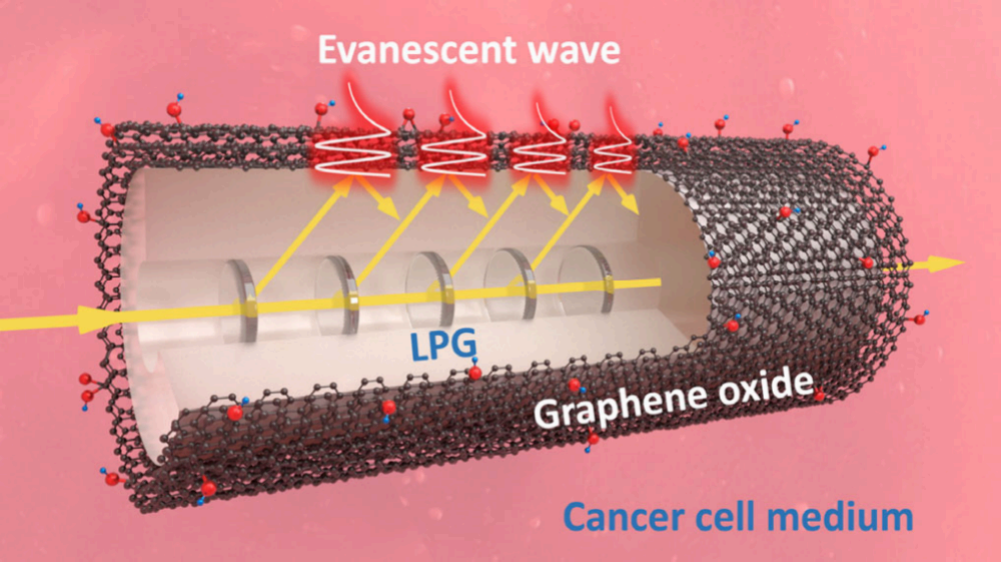
Schematic of GO-LPG for label-free detection.

## 2 Experimental Section

### 2.1 Materials and Characterization

Graphene oxide nanocolloids, sodium hydroxide (NaOH), (3-amino­propyl)­tri­eth­oxy­silane (APTES), Dulbecco’s Modified Eagle Medium (DMEM), and fetal bovine serum (FBS) were purchased from Sigma-Aldrich (UK). Acetone, ethanol, methanol, MCF-7 human breast cancer cells (ATCC), and CellTracker Green were purchased from Thermo Fisher Scientific Inc. (UK).

The surface morphological characterization of the GO overlay was investigated with an optical microscope (Olympus BX51, Leica Ltd., Germany), a scanning electron microscope (SEM, JSM-7100F LV, JEOL Ltd., Japan), an atomic force microscope (AFM, Bruker Dimension Icon, BRUKER Ltd., USA), and a DXR Raman spectrometer (Thermo Fisher Scientific Inc., UK). The fluorescent images of viable MCF-7 cells were obtained with an optical microscope (DMI8, Leica Ltd., Germany). The optical properties of GO-LPG were evaluated by using a super luminescent diode source (SLD, S5FC1550S-A2, Thorlabs Ltd., UK) and an optical spectrum analyzer (OSA, MS9740B, Anritsu Ltd., Japan).

### 2.2 Device Fabrication and Working Principle

The LPG, with a period of 400 μm and a length of 15 mm, was fabricated in a hydrogen-loaded single-mode fiber (SMF-28, Corning Inc., USA) using a continuous-wave and frequency-doubled Argon laser of 100 mW (Coherent Innova 90C, Coherent Inc., USA) at a wavelength of 244 nm and a point-by-point technique (with 50:50 ratio).^44^ The grating was subsequently annealed at 85 °C for 48 h to remove residual hydrogen and stabilize its optical properties.

When light was launched into the LPG, the fundamental core mode was coupled to forward-propagating cladding modes, resulting in several attenuation bands in the transmission spectrum. The wavelengths of the attenuation bands satisfy the phase matching condition:^45,46^

1 $$\lambda_{\mathrm{res}}=\left(n_{co}^{eff}-n_{cl,i}^{eff}\right)\Lambda$$

where *n*_*co*_^*eff*^ and *n*_*cl,i*_^*eff*^ are the effective RI of the core and *i*th cladding mode, respectively, and Λ is the grating period.

The transmission power *T* of the resonance is given by^45^

2 $$T=1-\sin^{2}\left(\kappa_{i}L\right)$$

where *L* is the grating length and *κ*_*i*_ is the coupling coefficient of the *i*th cladding mode. According to the mode coupling theory, the interaction between optical modes is proportional to their coupling coefficient. In cylindrical coordinates, the *κ* between two modes can be expressed as^47,48^

3 $$\kappa =\frac{\omega }{4P_{0}}\int_{\varphi =0}^{2\pi }\int_{r=0}^{\infty }\Delta \varepsilon \left(r,\varphi ,z\right)\psi_{vj}\left(r,\varphi \right)\psi_{\mu k}^{*}\left(r,\varphi \right)r\;dr\;d\varphi$$

where *P*_0_ is the power of the mode, ω is the FWHM of the grating profile, Δ*ε*(*r*, φ, *z*) is the permittivity variation, ψ­(*r*, φ) is the transverse field for the cladding mode, and *r* and φ represent the radial and angular field, respectively. The coupling coefficient is determined by the overlap integral of the core and cladding modes and on the RI modulation.^23,24,47,48^

It has been reported that GO has a complex RI *ñ* = *n* + i*k*,^49^ where *n* is the real part of the RI, primarily contributed by the π–π* transition, which dominates both reflection and transmission. The imaginary part, *k*, known as the extinction coefficient, is associated with the σ–σ* transition and governs light absorption and transmittance. The extinction coefficient has a strong influence on the optical properties.^50^ In addition, the extinction coefficient varies with the thickness of the GO overlay, thereby affecting its optical properties.^14,43,51,52^

For the GO nanocoated LPG, the sensing mechanism depends not only on the coupling coefficient between the core and cladding modes but also on the extinction coefficient of the nanocoating. Changes in the surrounding refractive index (SRI) can lead to fluctuations in the effective RIs of the cladding modes, resulting in a shift in the LPG resonance wavelength.^47,48,53^ Due to the complex RI of GO and changes in the electric field distribution, the coupling strength also changes, enabling intensity-based sensing mechanisms.^25–27,54,55^

### 2.3 Deposition of GO Nanosheets

We developed an in situ layer-by-layer deposition method by the conjunction of chemical bonding and physical adsorptions.^27^ The fiber device was initially cleaned with acetone (Figure 2a) and then immersed in a 1.0 M NaOH solution for 1 h, followed by rinsing with DI water and drying thoroughly (Figure 2b). For silanization, the OH-enriched device was incubated into a freshly prepared 5% APTES ethanol solution for 20 min at room temperature, followed by washing with ethanol and baking in an oven at 70 °C for 30 min to stabilize the Si–O–Si bonding (Figure 2c). Subsequently, the APTES-silanized device was immersed in 1 mL of a 0.1 mg/mL GO nanosheet suspension (Figures S1 and S2) contained in a custom-made mini-bath, which was heated to approximately 42 °C for 40 min (Figure 2d). The GO nanosheets were chemically bonded to the fiber surface through the reaction between the epoxy groups of GO and the amino groups on the APTES-silanized fiber surface (Figure 2e). Once the GO suspension was fully evaporated, a second cycle deposition was conducted by adding 1 mL of 0.1 mg/mL fresh GO suspension into the mini-bath, where the newly introduced GO nanosheets were physically adsorbed onto the previous GO overlay while the water solvent was gradually evaporating. After the second cycle deposition, the 2-cycle GO-coated LPG (2GO-LPG) was rinsed with DI water to remove any unbonded nanosheets and then baked in an oven at 35 °C for 12 h. Similarly, after the fourth cycle deposition, the 4-cycle GO-coated LPG (4GO-LPG) was washed with DI water to remove any nonadhered GO nanosheets and baked in an oven at 35 °C for 12 h.

**Figure 2:**
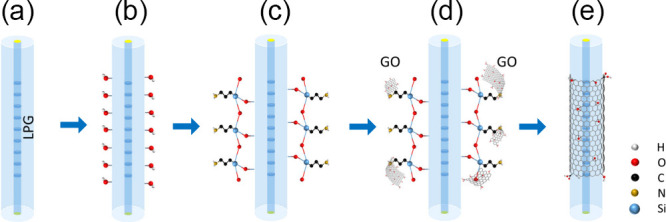
Schematic illustration of an optical fiber device functionalized by GO nanosheets. (a) Bare LPG. (b) Surface alkaline treatment. (c) Silanization by APTES. (d) GO deposition. (e) GO-coated LPG.

## 3 Results and Discussion

### 3.1 Surface Morphology Characterization

The surface morphologies of GO-coated fiber samples were characterized using an optical microscope, SEM, and AFM with the results shown in Figure 3a–d (for the 2-cycle coated sample) and Figure 3e–h (for the 4-cycle coated sample).

**Figure 3:**
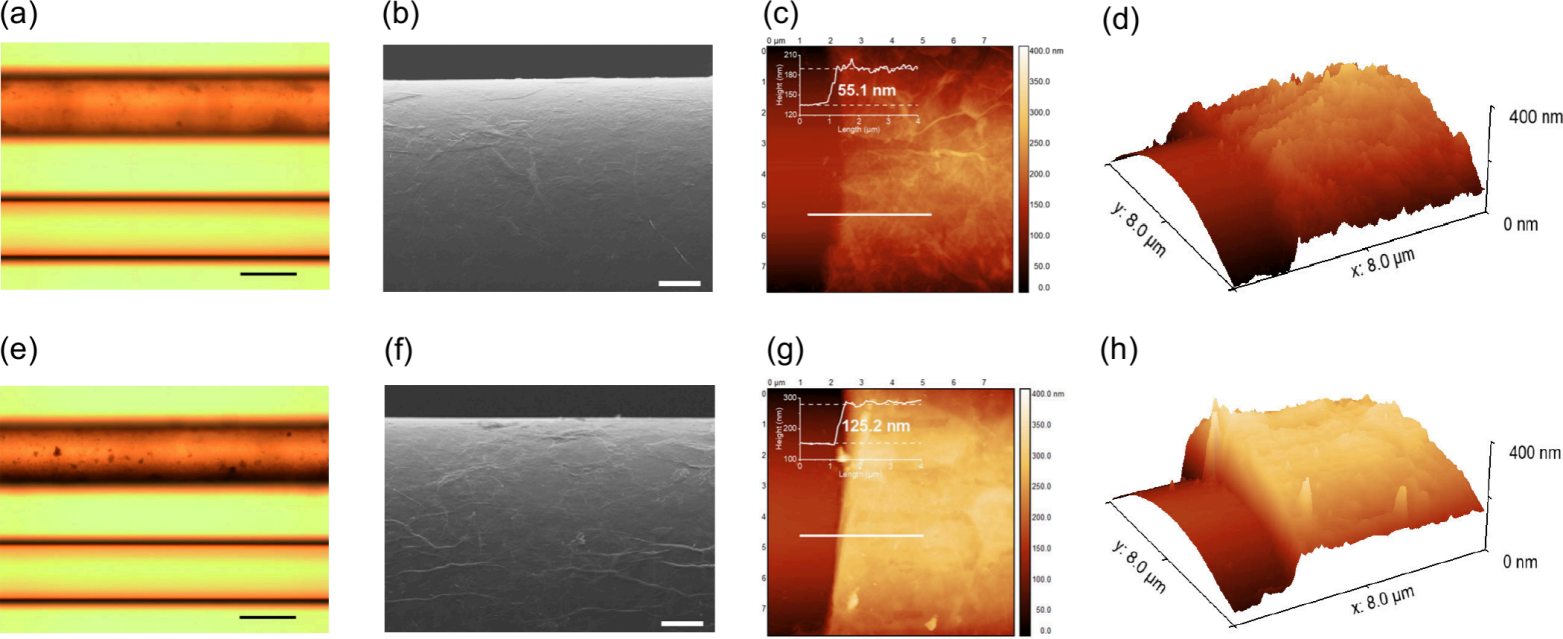
Surface morphological characterization of GO-coated fiber samples with 2-cycle coating (a–d) and 4-cycle coating (e–h). (a, e) Optical microscopy images (top: GO-coated fiber; bottom: bare fiber; scale bar: 100 μm). (b, f) SEM images (scale bar: 10 μm). (c, g) AFM images showing step boundaries between bare and GO-coated sections (inset: height profile of GO coating with precisely measured thicknesses). (d, h) 3D AFM images highlighting the GO step boundary.

Bare fiber samples exhibited a clear and transparent appearance in Figure 3a,e (bottom images). In contrast, the brown overlays were observed on both the 2-cycle and 4-cycle coated samples (top images), indicating the successful deposition of the GO overlay on the sample surfaces. Further SEM characterization (at 1500× magnification) revealed a homogeneous coating on the surfaces, where the 4-cycle coating (Figure 3f) displayed a more wrinkled texture compared to the 2-cycle coating (Figure 3b). AFM images clearly showed the step boundaries between bare and GO-coated sections, where the GO overlay thickness was precisely measured to be 55.1 nm (Figure 3c) and 125.2 nm (Figure 3g) for the 2-cycle and 4-cycle coated samples, respectively. Moreover, the 3D AFM images (Figure 3d,h) confirmed that the GO overlays were tightly wrapped around the entire cylindrical fiber surface.

As the Raman spectra show in Figure 4, the GO-coated samples exhibited three characteristic peaks of the G-band, D-band, and 2D-band, confirming the successful deposition of the GO overlay. The G-band at 1595 cm^–1^ corresponded to the first-order scattering of the E_2g_ phonon mode of sp^2^-hybridized carbon atoms. The D-band at 1330 cm^–1^ arose due to the structural defects and disorder, typically caused by the attachment of hydroxyl and epoxide groups on the basal plane and edges of the carbon lattice. The 2D-band was broader and weaker due to significant structural disorder and oxidation of the GO.^13^

**Figure 4:**
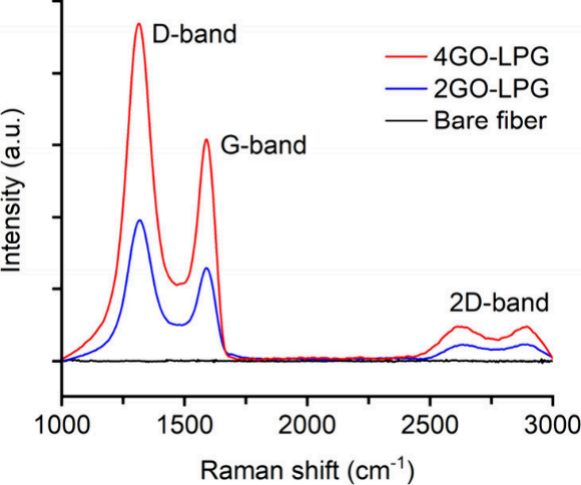
Raman spectra of the bare and GO-coated LPGs.

### 3.2 Effects of GO Thicknesses on Optical Properties

The effects of the GO thickness on optical properties were experimentally investigated by monitoring the transmission spectra of LPGs with varying GO thicknesses. An SLD light source was used to launch light into the grating device, and the transmission spectra were recorded using an OSA. To eliminate the cross-effects induced by bending and temperature, GO-LPG was placed in a custom-built container and kept straight while the external solution was applied. All measurements were conducted in a temperature-controlled room maintained at 21.0 ± 0.1 °C.

The transmission spectra of the LPG attenuation at the 1538 nm band were recorded before and after GO deposition (Figure 5a, all measured in water). The thin 2GO-LPG (with a 55.1 nm GO overlay) resulted in a 3.5 dB increase in peak intensity with a slight red-shift of 0.2 nm, while the thick 4GO-LPG (with a 125.2 nm GO overlay) led to a 6.3 dB increase in intensity along with a slight red-shift of 0.4 nm. These results indicate that the GO overlay enhances evanescent field interaction with the surrounding medium, resulting in a progressively intensity-dominant spectral response as the coating thickness increases.

**Figure 5:**
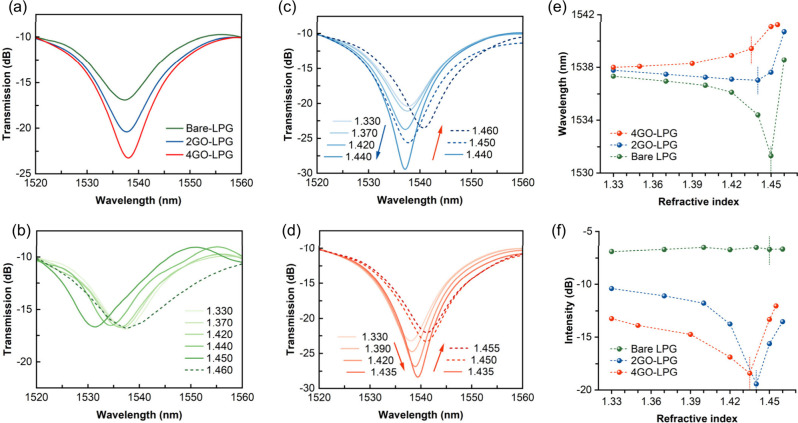
(a) Transmission spectra (measured in water) of bare and GO-coated LPGs. Thickness-dependent optical behaviors under varying SRI values for (b) bare LPG, (c) 2GO-LPG, and (d) 4GO-LPG. LPG resonance wavelength shift (e) and intensity change (f) vs SRI changes.

The optical properties of GO-LPGs with different GO overlay thicknesses were investigated under varying SRI values (Figure 5). The transmission spectra were monitored across a range of SRIs using index-matching gels with RIs from 1.330 to 1.460. For the bare LPG (Figure 5b), when the SRI increased from 1.330 to 1.450, the resonance showed a blue-shift of 6.01 nm, while the peak intensity presented a negligible change (Table 1). This behavior was consistent with that of conventional LPGs.^46,56^ For the thin-coated 2GO-LPG (Figure 5c), two distinct trends were observed in the attenuation band: (1) when the SRI increased from 1.330 to 1.440, a blue-shift of 0.75 nm was detected along with a 9.03 dB increase in intensity; (2) when the SRI increased from 1.440 to 1.460, the resonance exhibited a red-shift of 3.68 nm and a 5.91 dB decrease in intensity. In contrast, the thick-coated 4GO-LPG (Figure 5d) demonstrated a different behavior: (1) an opposite red-shift of 1.43 nm to a longer wavelength accompanied by a 5.18 dB increase in intensity as the SRI increased from 1.330 to 1.435; (2) a further red-shift of 1.80 nm but a 6.37 dB decrease in intensity when the SRI increased from 1.435 to 1.455.

**Table 1:** Wavelength Shift and Intensity Change under Different SRIs

	Sensor	SRI < TRI	SRI > TRI
Wavelength shift (nm)	Bare LPG	–6.01 nm	+7.24 nm
	2GO-LPG	–0.75 nm	+3.68 nm
	4GO-LPG	+1.43 nm	+1.80 nm
Intensity change (dB)	Bare LPG	–0.21 dB	–0.02 dB
	2GO-LPG	+9.03 dB	–5.91 dB
	4GO-LPG	+5.18 dB	–6.37 dB

As shown in Figure 5e,f, there are three different transition refractive index (TRI) points of 1.450, 1.440, and 1.435 for bare LPG, 2GO-LPG, and 4GO-LPG, respectively. The TRI point moved to a lower RI value as the GO thickness increased, which was consistent with previous findings.^48^

It was reported that the real part of GO’s complex RI ranged from 1.7 to 1.8, while the imaginary part lay between 0.3 and 0.4 in the 1550 nm wavelength region.^49^ The presence of a high refractive index (HRI) GO coating can induce a transition from cladding-guided modes to overlay-guided modes provided that the GO overlay is sufficiently thick. The transition can modify the effective RIs of cladding modes and consequently enhance the light–matter interaction between the optical device and the evanescent field.^47,48^ In addition, since GO presented a complex RI, the cladding modes were further influenced when the interaction reached a maximum at the light–matter interface with different GO thicknesses.

When the SRI was less than the TRI, only a slight blue-shift was observed for the thin-coated 2GO-LPG. This behavior could be attributed to the SRI increasing that facilitated the cladding mode transverse field profile to be stretched toward the HRI overlay, resulting in a reduction of the overlap integral between the core and cladding modes.^48^ In contrast, the thick-coated 4GO-LPG exhibited an immediate red-shift to an increasing of SRI, indicating that the presence of a thicker HRI overlay induced the strong changes in the cladding mode distribution, where the mode transition occurred from cladding-guided to overlay-guided modes. The transition significantly enhanced the interaction between the evanescent wave and the surrounding medium.

In the region where the SRI was lower than the TRI, the coupling coefficient increased as the SRI rose, which led to an increase in the resonance intensity of GO-coated LPGs. This behavior was consistent with the theoretical analysis in Section 2.2. Due to the complex RI of GO and the ongoing mode transition, both the coupling and extinction coefficients could be influenced. When the SRI approached the TRI, GO-LPGs exhibited higher sensitivities of 283.95 and 102.47 dB/RIU for the thin- and thick-coated LPGs, respectively (Table 2). When the SRI exceeded the TRI, the overlay-guided modes became dominant, and the extinction coefficient changed dramatically, with the light interaction reaching its maximum at the interface. This resulted in maximum RI sensitivities of −295.65 and −318.55 dB/RIU for 2GO-LPG and 4GO-LPG, respectively.

**Table 2:** Intensity-Based RI Sensitivities of GO-LPGs in Different RI Regions

RI Region	2GO-LPG	4GO-LPG
1.330–1.420	+37.22 dB/RIU	+40.43 dB/RIU
1.420–1.440	+283.95 dB/RIU	+102.47 dB/RIU
1.440–1.460	–295.65 dB/RIU	–318.55 dB/RIU

### 3.3 Breast Cancer Cell Culture and Incubation

MCF-7 breast cancer cells (Figure 6a for the bright-field image) were seeded into a 6-well plate (Figure 6b) at the following cell numbers counted with a traditional hemocytometer: (1) 0 (control, DMEM only), (2) 1000, (3) 10,000, (4) 100,000, and (5) 500,000 cells per well, each in 5 mL of DMEM supplemented with 5% FBS. The plate was incubated for 72 h under standard growth conditions (37 °C, 5% CO_2_). After the 72 h incubation, the cell culture media were carefully collected into sterile Eppendorf tubes (Figure 6c), with the initial concentrations corresponding to 0, 2 × 10^2^, 2 × 10^3^, 2 × 10^4^, and 1 × 10^5^ cells/mL.

**Figure 6:**
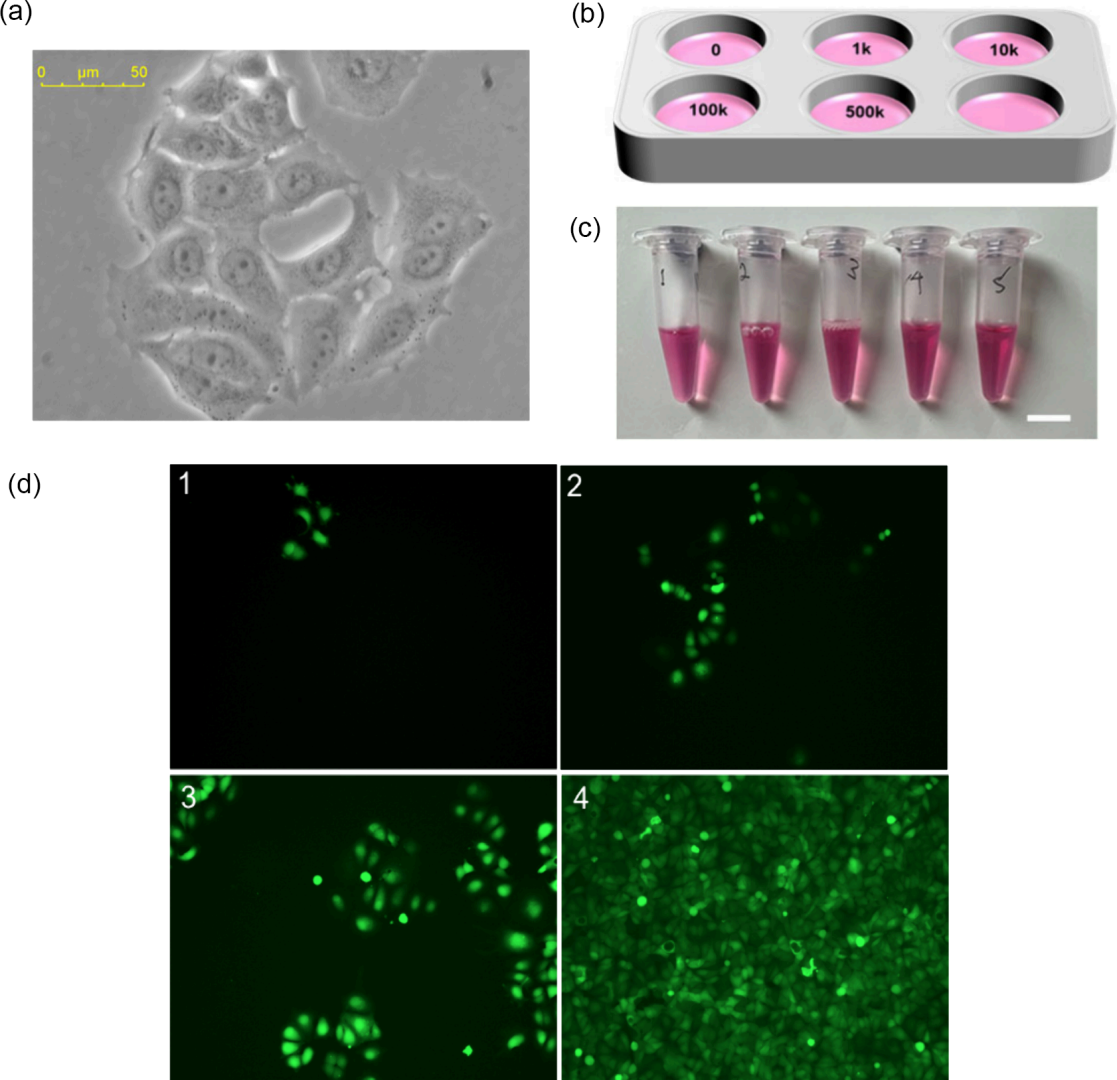
(a) Bright-field image of viable MCF-7 cancer cells. (b) Schematic of the MCF-7 cell culture setup. (c) Collection of conditioned culture media (scale bar: 10 mm). (d) Fluorescent images of MCF-7 cells labeled with CellTracker Green at initial seeding densities of (1) 1000, (2) 10,000, (3) 100,000, and (4) 500,000 cells per well.

To confirm the cell density, the cells were incubated with CellTracker Green (0.1 nM) for 15 min and then imaged with a Leica DMI8 microscope with a 10× objective lens (Figure 6d). A progressive increase in the fluorescence intensity was observed with increasing cell number, indicating good viability and effective staining of the cultured cells. Additionally, the cells were strained with crystal violet solution to provide additional confirmation of the cell density (Figure S3).

### 3.4 Label-Free Detection via Culture Media

The proposed sensor was utilized for the label-free detection via RI sensing of cancer cell media with initial concentrations of 0, 2 × 10^2^, 2 × 10^3^, 2 × 10^4^, and 1 × 10^5^ cells/mL, respectively. The cell media were mixed with a sucrose solution (RI = 1.455), which served as a high sensitivity RI buffer. It is important to note that the collected media contained no cells but only the metabolic byproducts secreted by the cancer cells during the culture period.

The 4GO-LPG was employed to detect the conditioned media with the experimental setup in Figure 7a. An SLD source launched light into the fiber sensor, while the transmission spectra were monitored by an OSA. The fiber sensor was mounted in a custom-built container, which was fabricated from Teflon with a precision laser-cut groove along the centerline to hold the fiber sensor in a straight configuration. A pipette was used to manually introduce biosamples and rinse the sensor during the measurements. As shown by the transmission spectra recorded in Figure 7b, a significant increase in resonance intensity of 2.95 dB was observed from the medium with increasing initial concentration from 0 to 1 × 10^5^ cells/mL. The limit of detection (LOD) was determined to be 270 cells/mL, which was calculated based on the following equation:^57^

4 $$x_{\mathrm{LOD}}=f^{-1}\left({\mathrm{y̅}}_{\mathrm{blank}}+3\sigma_{\max}\right)$$

where *x*_*LOD*_ is the limit of detection, *y̅*_*blank*_ is the mean value of the blank sample, and σ_*max*_ represents the maximum standard deviation. The quantitative data (Figure 7c) were expressed as the mean ± standard deviation, with each data point averaged from a minimum of three independent measurements.

**Figure 7:**
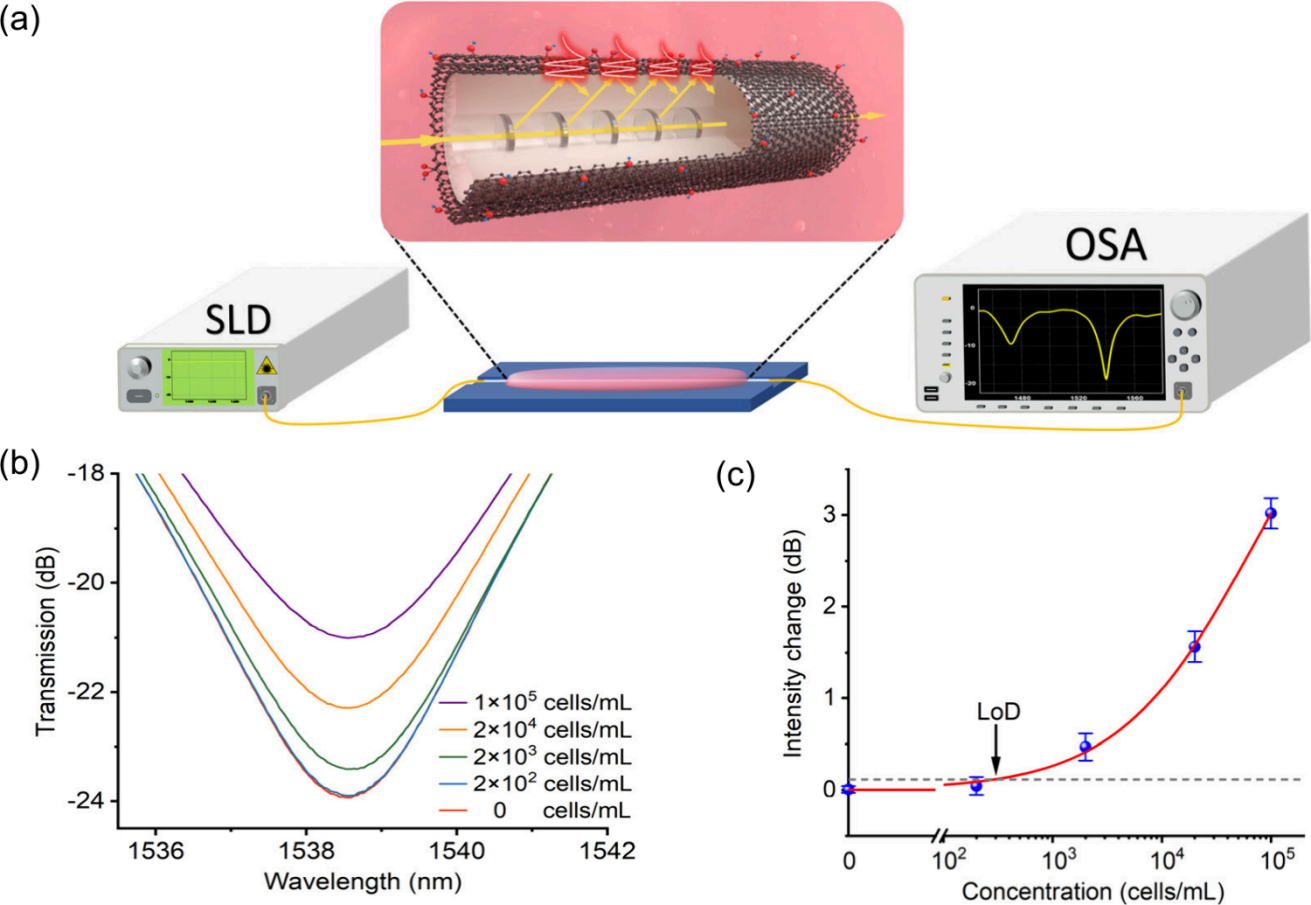
(a) Schematic illustration of the GO-LPG sensing system. (b) Transmission spectra corresponding to different cancer cell medium concentrations. (c) Intensity changes of attenuation band against different cell medium concentrations.

## 4 Conclusions

We have developed a GO-functionalized LPG sensor for the label-free detection of the presence of breast cancer cells through metabolic byproducts. GO nanosheets were uniformly deposited onto LPGs using an i-LbL assembly technique, yielding homogeneous nanocoatings with precisely controlled thicknesses of 55.1 and 125.2 nm. The effects of the GO overlay thickness on the optical properties of the sensors were experimentally investigated and characterized. The GO-LPG sensor demonstrated detection of MCF-7 cell density with ultrahigh sensitivity, achieving an LOD as low as 270 cells/mL. This label-free, noninvasive bionanophotonic sensing platform holds strong promise for early cancer diagnostics, biomedical detection, and therapeutic monitoring.

## Supplementary Material



## Notes

UV–vis absorption of GO nanosheet suspension, AFM image of GO nanosheets, and image of MCF-7 cancer cells stained with crystal violet (PDF)
