# Supplementary material for: Graphene Oxide-Functionalized Optical Sensor for Label-Free Detection of Breast Cancer Cells
Source: ACS Appl Nano Mater. 2025 Aug 18;8(34):16770–8. doi: 10.1021/acsanm.5c02864 (PMC12403181; doi:10.1021/acsanm.5c02864)
Supplement: Supplementary file 1 [file an5c02864_si_001.pdf]

Supporting Information

Graphene Oxide-Functionalized Optical Sensor for Label-Free Detection of Breast Cancer Cells

Jiaxing Sun[a], Hanlin Jiang[a], Kartikey J. Chavan[a], Amanda S. Coutts[b], Xianfeng Chen[a*]

[a] *Department of Physics, School of Science and Technology, Nottingham Trent University, Nottingham NG11 8NS, UK*

[b] *John van Geest Cancer Research Centre, Department of Biosciences, Nottingham Trent University, Nottingham NG11 8NS, UK*

[*] *Corresponding author: xianfeng.chen@ntu.ac.uk (Xianfeng Chen)*

Figure S1 reveals the UV-Vis absorption spectrum of GO nanosheets suspension after the sonication. The shoulder of the absorption spectrum, located at 300 nm, is attributed to the $\pi \rightarrow \pi^*$ transitions of C=O bonds, which is consistent with the presence of oxygen functional groups on GO nanosheets. The GO suspension (inset) exhibits a dark color due to its broad absorption band spanning the visible spectrum.

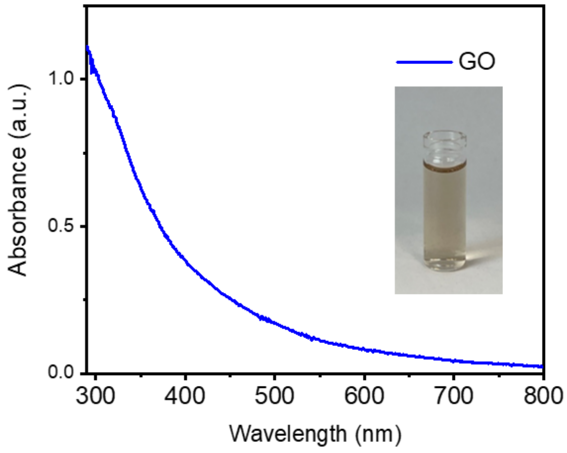

Figure S1. The UV-Vis absorption spectrum of GO suspension (inset: photo of GO suspension).

The exfoliated GO nanosheets were characterized with AFM for identifying their thickness and size (**Figure S2**). The thicknesses of single layer GO nanosheets were measured as 1.0

nm. Smaller GO nanosheets exhibited lateral sizes between approximately 0.7~1.0 µm, while larger nanosheets extended from 2.5 µm up to around 6.0 µm. This variation indicated the presence of both small fragments and relatively large and intact sheets.

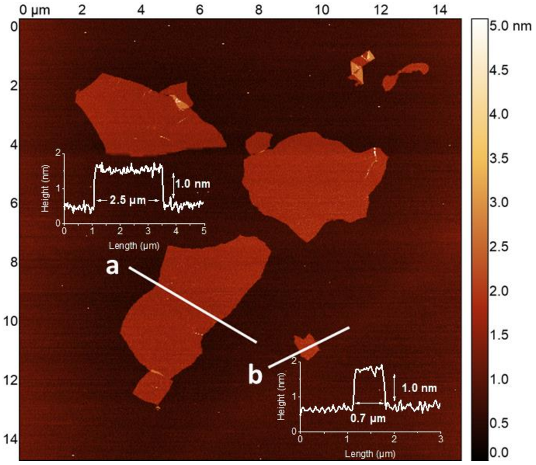

Figure S2. AFM image of single layer GO nanosheets. Inset: height profiles of two GO nanosheets measured along two white lines: (a) larger GO nanosheet, (b) smaller GO nanosheet.

To further confirm the cell density, MCF-7 cancer cells were stained with crystal violet solution for 10 min and subsequently imaged using a camera (**Figure S3**), which revealed differences in cell density across the wells.

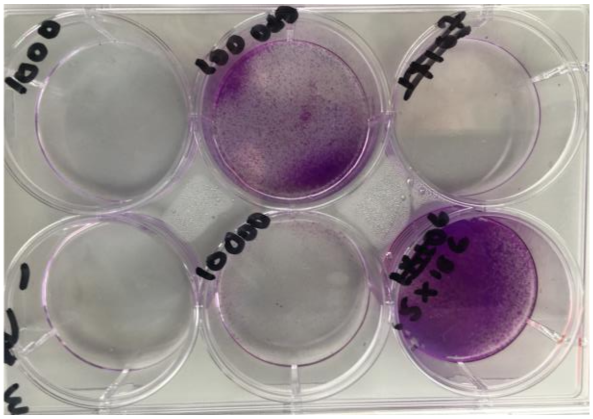

Figure S3. The images of MCF-7 cancer cells stained with crystal violet, highlighting the differences in cell density across wells.
